# Contribution of health workforce to health outcomes: empirical evidence from Vietnam

**DOI:** 10.1186/s12960-016-0165-0

**Published:** 2016-11-16

**Authors:** Mai Phuong Nguyen, Tolib Mirzoev, Thi Minh Le

**Affiliations:** 1 Vietnam Ministry of Health, 138A Giang Vo street, Ba Dinh district, Hanoi, Vietnam; 2 Nuffield Centre for International Health and Development, Leeds Institute of Health Sciences, University of Leeds, Charles Thackrah Building, 101 Clarendon Road, Leeds, LS2 9LJ United Kingdom; 3 Hanoi University of Public Health, 1A Duc Thang, Duc Thang ward, Bac Tu Liem district, Hanoi, Vietnam

**Keywords:** Health workforce, Human resources for health, Health outcomes, Infant mortality, Under-five mortality, Life expectancy, Vietnam, Asia

## Abstract

**Background:**

In Vietnam, a lower-middle income country, while the overall skill- and knowledge-based quality of health workforce is improving, health workers are disproportionately distributed across different economic regions. A similar trend appears to be in relation to health outcomes between those regions. It is unclear, however, whether there is any relationship between the distribution of health workers and the achievement of health outcomes in the context of Vietnam. This study examines the statistical relationship between the availability of health workers and health outcomes across the different economic regions in Vietnam.

**Methods:**

We constructed a panel data of six economic regions covering 8 years (2006–2013) and used principal components analysis regressions to estimate the impact of health workforce on health outcomes. The dependent variables representing the outcomes included life expectancy at birth, infant mortality, and under-five mortality rates. Besides the health workforce as our target explanatory variable, we also controlled for key demographic factors including regional income per capita, poverty rate, illiteracy rate, and population density.

**Results:**

The numbers of doctors, nurses, midwives, and pharmacists have been rising in the country over the last decade. However, there are notable differences across the different categories. For example, while the numbers of nurses increased considerably between 2006 and 2013, the number of pharmacists slightly decreased between 2011 and 2013. We found statistically significant evidence of the impact of density of doctors, nurses, midwives, and pharmacists on improvement to life expectancy and reduction of infant and under-five mortality rates.

**Conclusions:**

Availability of different categories of health workforce can positively contribute to improvements in health outcomes and ultimately extend the life expectancy of populations. Therefore, increasing investment into more equitable distribution of four main categories of health workforce (doctors, nurses, midwives, and pharmacists) can be an important strategy for improving health outcomes in Vietnam and other similar contexts. Future interventions will also need to consider an integrated approach, building on the link between the health and the development.

**Electronic supplementary material:**

The online version of this article (doi:10.1186/s12960-016-0165-0) contains supplementary material, which is available to authorized users.

## Background

During the last few decades, there has been an increasing interest in exploring relations between availability of human resources for health and health outcomes [1]. Given that population’s health outcomes are a product of complex and interdependent interventions, disentangling and weighting this relation can be useful for informing policy reforms.

Vietnam is a lower-middle income country with a population of 89.71 million and income per capita of $1911 in 2013 [2, 3]. Since the implementation of an open-door policy in 1986, the country’s economy has been steadily increasing at the annual rate of about 6–7% GDP 3 [4–11]. Vietnam is divided into six economic regions: *Red River Delta*, *Northern Midland and Mountain area*, *North Central area and Central Coastal area*, *Central Highlands*, *Southeast*, and *Mekong River Delta*.^1^ These six regions differ significantly in terms of their socioeconomic development. The Southeast is the richest region with the annual income per capita of approximately 2100 USD, followed by the Red River Delta. The Northern midlands and Mountain areas, as well as the Northern Central and the Central Coastal areas have the lowest per capita incomes of 875 USD and 1000 USD, respectively [11].

In terms of the country’s health outcomes, average life expectancy at birth (LE) has seen a steady rise over the last decade, reaching 73.1 years in 2013. The infant mortality rate (IMR) declined from 36.6 per 1000 live births in 1999 to 15.3 in 2013. The under-5-year-old mortality rate (U5MR) has also declined steadily each year, falling from 58 to 23.1 in 2013 [4–11]. The maternal mortality ratio also declined to 69 per 100 000 live births in 2012 from 233 per 100 000 live births in 1990. However, the basic health indicators also show the regional disparities. In 2013, while the average life expectancy in the Southeast had reached 75.7 years, it only reached 69.7 years in the Central Highlands. The Vietnam Millennium Development Goals report in 2013 and 2015 [12, 13] also mentioned that despite the government’s efforts to address the socioeconomic gap between those economic regions, the disparity of infant and under-five mortality rate still exists between regions. The highest mortality rates are seen in mountainous and disadvantaged regions such as Central Highlands which had an IMR 2.7 times higher than in the Southeast in 2009, and this gap remains high at approximately two times in 2013 [11].

The economic growth has created a momentum for Vietnam to invest in its healthcare system. Overall national health expenditure has risen from being only 2.9% of the state budget in 2005 to becoming 6% in 2012, with all six economic regions experiencing the same trend. Given the rise of total state budget during that period, national health expenditures have increased in real terms more than three times [11]. As a result, investment in health workforce, one of the most important components of health care system in Vietnam, has been gradually increasing over the last decade, reflected in growing numbers of all categories of health professionals in all country’s six regions. In Vietnam, health professionals or health workers are defined as those who study, advise on, or provide health services and include doctors, nurses, midwives, and pharmacists [14]. However, health workers are disproportionately distributed among economic regions. For example, the density of doctors in the Southeast region is 7.8 per 10 000 inhabitants, compared with 5.7 in Central Highlands. Meanwhile, the Southeast region, being a richer region, attracts nearly three times of the total number of health workers in the Central Highlands [15].

In this context, the importance of health workers cannot be underestimated. A number of policies have been enacted to develop and retain health workers in disadvantaged areas which included increased remuneration, allowances, and educational and promoting opportunities [14]. In addition, the program that brings skilled health professionals to provide on-the-job-training to health workers in disadvantaged areas has enhanced the capacity and quality of human resources. Furthermore, the deployment and training of ethnic minority midwives has been recognized as an interim solution to address the lack of health workers to provide maternal and child care in remote areas [16]. Along with these policies, the education and training of health workers are now more oriented to addressing the health system’s needs. Gradual development of family doctors has contributed to the improved distribution of health professionals. In other words, community-based care became a fundamental element of the development of human resource policies to attract and retain adequate number of health workers and ensure their appropriate distribution.

However, the imbalanced distribution of human resources across economic regions still remains a critical issue to address, to help the country’s health system achieve its goals of equity, efficiency, and quality. Several reasons of the maldistribution were documented from both demand and supply sides such as low production capacity, restricted capacity for employment of graduates in remote areas, low pay in the public sector, poor services in remote areas, financial barriers, and cultural factors [17–20]. One important question of interest to the national policymakers is how the availability and distribution of heath workforce interacts with, and contributes to, improved health outcomes. In this longitudinal study, we statistically explore and weight the link between the availability of health workers and health outcomes based on the nationally representative data in Vietnam. We do so by applying the principal component analysis (PCA) to estimate the impact of four categories of health workers (doctors, nurses, midwifes, and pharmacists) on health outcomes.

We believe this analysis should be of interest to a broad range of national and international stakeholders, for example policymakers in countries with similar contexts to Vietnam, and researchers who are interested in improving their understanding of relationship between health workers and health outcomes. Our analysis can also inform future policies addressing the maldistribution of health workers. Furthermore, it can provide evidence for designing strategies for improving health outcomes in the longer term in Vietnam and other similar contexts.

## Methods

In this paper, we report analysis of the relationship between availability of health workers and key health outcomes in Vietnam. As mentioned earlier, the health workers in Vietnam are defined according to the WHO classification of health professionals which is based on International Standard Classification of Occupations (ISCO, 2008 revision). In this paper, we use terms health professionals and health workers interchangeably and both to mean those who study, advise on, or provide health services [21]. The WHO classification differentiates between health professionals (e.g., doctors, and nurses), health associate professionals (e.g., community health workers), personal care workers (e.g., health care assistants), and health management and support personnel (e.g., health managers). While we recognize the potential importance of all above categories, in this paper, we specifically focus on exploring the impact of four categories of health professionals: doctors, nurses, midwives, and pharmacists. In our experience, these four generic categories can be found in most health systems, thus contributing to relevance of our results to different contexts.

Our research covered only the public sector. This is because Vietnam’s healthcare system is dominated by the public sector. The private health sector in Vietnam is generally small, for example in 2013 accounts for only 3.34% of total healthcare beds [22], and the data on the private workforce was sparsely reported during the time period covered in our study (2006–2013).

### Data collection and quality

We constructed a panel data, which is a collection of economic and health-related features of each economic region over 8 years, to examine the determinants of health outcomes. We used health outcomes as dependent variables and economic and health workforce as our main explanatory variables. Our data includes a panel of six economic regions of Vietnam (Red River Delta, North Midlands and Mountain areas, North Central and Coastal area, Central Highlands, Southeast area, and Mekong River Delta), sourced from Statistics Yearbooks by Vietnam General Statistics Office and the Ministry of Health covering an 8-year period from 2006 to 2013. The data on life expectancy at birth, infant mortality rate, and under-five mortality rate were obtained from the Population and Housing Census 2009 for the year 2009 and from annual surveys on Population Change and Family Planning for the remaining years. These indicators are published and nationally representative. For the period before 2009, the mortality rates were obtained directly from civil registration and government reporting systems in Vietnam [23]. The limitation of this data is that the death rate may be under-reported, leading to unreliable data. However, the mortality rates were reconstructed by BRASS methods, estimating child mortality from information on aggregate numbers of children ever born and children still alive (or dead) reported by women classified by age group, to indirectly estimate measures of mortality level in 2009 [24, 25]. This method provided more accurate calculation and estimates of the mortality indicators and minimized the number of missing death counts from surveys.

Data on one of our health outcome variables, i.e., the life expectancy of each region was unfortunately not available in 2007. As the data are specific to each region which is evidently correlated to each other as shown by our cross-sectional dependence test, we approximated the missing values in 2007 by computing the mean of life expectancy in 2006 and 2008. We then calculated the density of doctors, nurses, midwives, and pharmacists per 10 000 inhabitants by simply dividing the corresponding variables to the total regional population based on data available from Vietnam and Health Statistics Yearbooks from 2006–2013 [22, 26–32].

### Variables and econometric modeling

In developing countries, life expectancy at birth and mortality rates are typically used as indicators of health outcomes and measures of the health status of the population [33]. We chose life expectancy at birth, infant mortality rate, and under-five mortality rate as dependent variables which not only provide a general picture but also help to evaluate the health performance of the economic regions over time. In addition, under-five mortality rate (U5MR) and infant mortality rate (IMR) are the two important indicators of the progress towards the achievement of United Nations Millennium Development Goals. While the IMR assesses more pre-natal health conditions, U5MR reflects more nutrition and nursing care conditions. The maternal mortality rate is another important dependent variable, but unfortunately, the data was incompletely recorded for our study periods.

We selected the explanatory variables based on the literature and data availability of Vietnam. In existing literature, healthcare, education, and socioeconomic environments are determined as main determinants of health outcomes [34–37]. Health workers are one of three most important inputs of a health system [38]. The densities of four main categories of health workers (doctors, nurses, midwives, and pharmacists) are included in the model as predictors for health outcomes. In Vietnam, there are also assistant doctors whose qualifications are recognized as a college degree. They assist doctors to care for patients, and their job is similar to the nurses. Therefore, we combined the number of nurses and assistant doctors in estimating the density of nurses. Conversely, we did not aggregate the number of midwives and nurses because midwives’ job and qualification are distinct from those of nurses (for example, nurses are responsible for provision of all health services whereas midwives are primarily responsible for maternal health services such as obstetric care). Pharmacists (including high, middle, and elementary degree) are the health professionals most accessible to the public, and the role of pharmacists is not only to provide an accurate medication but also to advise patients on the appropriateness of different medicines at the time of dispensing them.

Furthermore, to account for major socioeconomic determinants of health outcomes, we included the following explanatory variables. Income per capita and poverty rate are included as proxies for economic infrastructure. Although these two variables are correlated, the first captures the level of average income while the second describes the distribution of low income across regions. The higher rate of poverty is likely to lead to higher mortality rate [17]. These variables also capture several distal factors (nutrition, safe water provision, sanitation, housing, urbanization) that affect both mortality rates and life expectancy [39].

As there is extensive evidence of the association between parental education and child health [37], we included the adult illiteracy rate as a proxy variable for parental education, due to the unavailability of female illiteracy rate by regions over the 8-year period. Population density of the regions is also included as an explanatory variable, accounting for environmental factors which are likely to impact on the health service coverage and spread of diseases [28] thus influencing health outcomes.

The dependent and explanatory variables are summarized in Table 1.

**Table 1:** Definitions and measurements of dependent and explanatory variables

Variables	Unit	Label	Measurements
Dependent			
Life expectancy at birth	Year	LE	The number of years newborn children would live if subject to the mortality risks prevailing for the cross-section of population at the time of their birth
Infant mortality rate	Percentage	IMR	Number of deaths under 1 year of age per 1 000 live births
Under-five mortality rate	Percentage	U5MR	Number of deaths under age 5 per 1 000 live births
Explanatory			
Density of doctors	Number	DOC	Number of working doctors per 10 000 population
Density of nurses	Number	NUR	Number of working nurses per 10 000 population
Density of midwifes	Number	MID	Number of working midwives per 10 000 population
Density of pharmacists	Number	PHA	Number of working pharmacists (including high, middle and elementary degree) per 10 000 population
Income per capita	Million VND	IPC	Total income of households in reference year divided by their headcounts
Regional population density	Person/km^2^	RPD	Average number of people per square kilometer
Poverty rate	Percentage	PR	The proportion of population living below the poverty line (calculated using World Bank methodology for developing countries)
Adult illiteracy rate	Percentage	IR	The proportion of illiterate persons aged over 15, as a percentage of over total population aged over 15

First, we built a panel model of which health outcomes depend on workforce variables, controlled socioeconomic variables and some unobservable factors

$$ {y}_{it} = {\beta}_1\mathrm{Doc} + {\beta}_2\mathrm{N}\mathrm{u}\mathrm{r} + {\beta}_3\mathrm{M}\mathrm{i}\mathrm{d} + {\beta}_4\mathrm{P}\mathrm{h}\mathrm{a} + {\beta}_5\mathrm{R}\mathrm{p}\mathrm{d} + {\beta}_6\mathrm{I}\mathrm{p}\mathrm{c} + {\beta}_7 \Pr +{\beta}_8\mathrm{I}\mathrm{r}+{\beta}_0\mathrm{Time}+{c}_i+{u}_{it} $$

where

- *y*_*it*_ is the health outcome indicator which takes turn to be IMR, U5MR, and LE; the subscript *i* represents the region and *t* the time.
- *β*_*k*_, *k* = 1,…,8, are the coefficients showing the impact of each explanatory variable on dependent variables
- Time is a dummy variable which is equal to 0 if the observations are of the year 2006, 2007, and 2008, and equal to 1 if observations are from 2009 to 2013. This variable is created to account for the application of a new method to measuring the mortality rate and life expectancy, as we explained in the data section.
- {*u*_*it*_} is error term *independent* to the explanatory variables. Those errors account for unobserved factors that influence health outcomes—for example investment in healthcare infrastructure, population’s education, healthcare policies and health management, and environments—which can vary across time and the regions.
- *c*_*i*_ is the unobservable characteristics of each region that do not change over time such as geography and culture.

It is well known that nurses, midwives, and pharmacists do not work separately but in conjunction with doctors; the income per capita, poverty rate, and adult illiteracy rate as proxies for socioeconomic variables are often related to each other. Given those entangled relationships among explanatory variables, there could be many pairwise correlations among the explanatory variables (multicollinearity) which violate the conventional assumption on independency among explanatory variables in standard panel analyses thereby resulting in volatile and unreliable estimates. We therefore needed to test for the multicollinearity among the explanatory variables by performing variance inflation factors to quantify how much the standard errors of estimated coefficients is inflated when multicollinearity exists as shown in Additional file 1: Appendix 1.

As the test result shows evidence of multicollinearity, we applied the principal component analysis method to analyze our model. This technique transforms a number of highly correlated variables into a smaller number of uncorrelated variables called principal components. Each principal component corresponds to a linear combination of variables. The first principal component accounts for as much of the variability in the data as possible, and each succeeding component accounts for as much of the remaining variability as possible. The analysis was performed in four main steps. First, we standardized variables as in usual practice to make the PCA robust to their different measurements. Second, we extracted the principal components corresponding to the highest eigenvalues of the explanatory variable matrix [40]. In the third step, we ran an appropriate regression of the standardized dependent variable *y*_*it*_ on the principal component of explanatory variables which accounts for specific features of our panel, i.e., cross-sectional dependence and serial correlated errors of order 1. In the last step, we recovered the interested coefficients in our model. More details of each step can be found in Additional file 1: Appendix 2.

### Specification tests

We employed the Frees (1995, 2004) test for cross-sectional dependence in our panel. The result implied a presence of cross-sectional dependence among the six regions, as shown in Additional file 1: Table A5 of Appendix 3. We also included the Wooldridge test for serial correlation among error terms in the Additional file 1: Table A5 of Appendix 3. The test provided some evidence for serial correlation of order 1. Therefore, we employed the PCR panel regression with corrected standard errors, taking into account those cross-sectional dependency and serial correlation as recommended by Beck and Katz [41]. This is an alternative to feasible generalized least square for fitting a linear model that presents heteroskedastic and contemporaneously correlated across panel and serially correlated errors over time, like in our case. This method corrects the standard errors of estimates by taking into account the disturbance covariance matrix across individuals in our panels, which otherwise are usually unacceptable optimistic if being estimated by feasible generalized least square method.

## Results

In presenting the results, we first provide an overview of the availability of health workers in Vietnam between 2006 and 2013. This is followed by details of the descriptive statistics of independent and explanatory variables and then exploration of impacts of availability of health workers on key health outcomes.

### Overview of the workforce from 2006 to 2013

**Fig. 1:**
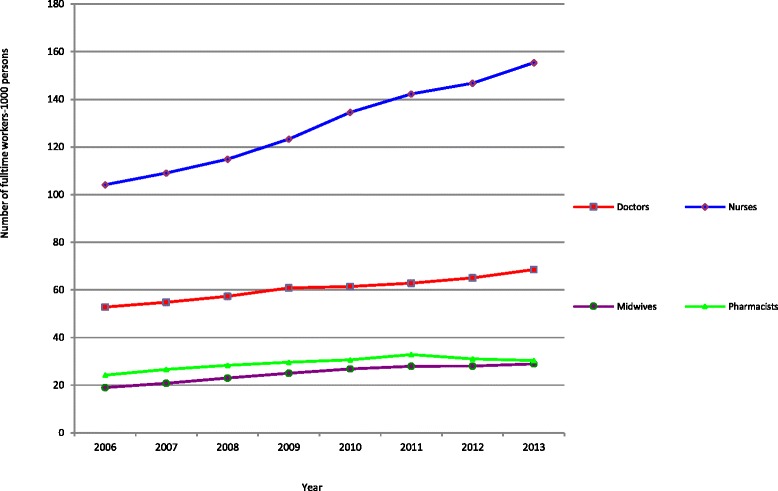
Four main categories of health workers during 2006–2013

Figure 1 below shows trends in availability of the total number of doctors, nurses, midwives, and pharmacists in Vietnam between 2006 and 2013.

As shown in Fig. 1, the numbers of doctors, nurses, midwives, and pharmacists have been rising over the last decade. However, there are notable differences between the different categories. While the number of nurses rocketed from about 110 000 in 2006 to about 155 000 in 2013, increases in availability of three other categories of health workers have been more gradual. While the overall number of pharmacists had risen between 2006 and 2013, we also found a slight reduction in their total numbers between 2011 and 2013.

### Descriptive statistics of independent and explanatory variables by regions 2006–2013

As shown in Table 2, Central Highlands has the worst indicators of health outcomes, followed by Northern Midland. On the other hand, Southeast and Red River Delta regions have some of the best indicators. The situation is similar to the regional distribution of doctors and income per capita. Disparity of health indicators among these regions is substantial. For example, life expectancy in the Highlands region is 5 years lower than that in the Southeast region. The Red River Delta (including the capital Hanoi) and the Southeast regions have the highest density of doctors and pharmacists, while the Highlands region has the highest density of midwives.

**Table 2:** Means and standard deviations of independent and explanatory variables by regions

Variables	Red River Delta	North Midland & Mountain areas	North Central & Coastal area	Central Highlands	Southeast	Mekong River Delta
LE	74.39 (0.23)	70.24 (0.53)	72.23 (0.69)	69.50 (0.59)	75.40 (0.31)	74.17 (0.46)
IMR	11.78 (0.88)	23.7 (1.53)	17.84 (2.00)	26.21 (1.80)	9.48 (0.78)	12.31 (1.14)
U5MR	17.61 (1.36)	36.00 (2.40)	26.86 (3.07)	39.95 (2.83)	14.15 (1.13)	18.44 (1.75)
DOC	9.56 (0.55)	6.84 (0.71)	6.33 (0.30)	5.16 (0.44)	7.08 (0.58)	5.16 (0.63)
NUR	14.92 (2.39)	19.52 (2.78)	14.39 (1.22)	12.94 (1.94)	13.49 (2.31)	12.62 (1.90)
MID	2.14 (0.29)	3.32 (0.41)	3.14 (0.32)	3.31 (0.37)	2.69 (0.39)	2.67 (0.43)
PHA	4.26 (0.36)	3.04 (0.47)	2.69 (0.21)	2.17 (0.39)	2.97 (0.19)	3.94 (0.50)
IPC	11.63 (2.56)	6.76 (1.01)	7.67 (1.40)	8.35 (1.35)	17.27 (2.05)	12.01 (4.26)
RPD	938.93 (23.44)	117.31 (2.33)	197.58 (2.61)	94.58 (3.90)	603.86 (39.77)	424.66 (4.43)
IR	3.00 (0.93)	13.03 (2.34)	6.40 (0.73)	10.84 (2.12)	4.60 (1.56)	8.51 (1.58)
PR	7.83 (1.71)	25.99 (2.34)	18.86 (2.65)	20.7 (2.57)	2.15 (0.71)	11.51 (1.28)

Note: The number in parentheses is the standard deviation

### Impact of availability of health workforce on health outcomes

Regression models with standardized dependent and explanatory variables provided the results, which are shown in Table 3.

**Table 3:** The impact of health workforce on health outcomes

Standardized explanatory variables	Model 1: IMR	Model 2: U5MR	Model 3: LE
β_1_ (DOC)	−0.15** (0.05)	−0.15** (0.05)	0.17** (0.04)
β_2_(NUR)	−0.13* (0.05)	−0.13* (0.05)	0.14** (0.04)
β_3_(MID)	−0.24** (0.07)	−0.24** (0.07)	0.26** (0.06)
β_4_ (PHA)	−0.43** (0.16)	−0.43** (0.16)	0.45** (0.14)
β_5_ (IPC)	−0.12* (0.05)	−0.13* (0.05)	0.14 ** (0.04)
β_6_(RPD)	−0.12* (0.05)	−0.12* (0.05)	0.14** (0.04)
β_7_ (PR)	0.02 (0.06)	0.02 (0.06)	−0.03 (0.05)
β_8_(IR)	0.16** (0.06)	0.16** (0.06)	−0.18** (0.04)
Number	48	48	48
Adjusted *R* ^2^	0.52	0.53	0.64
Wald test	–	–	–
Pro > *F*	<0.00	<0.00	<0.00

The number in parentheses is the standard error

*Significant at the 5% level

**Significant at the 1% level

The coefficients of explanatory variables to dependent variables before standardization are recovered and presented in Tables 4 and 5.

**Table 4:** Estimated impacts of increasing one health worker per 10 000 population on health outcomes in each region

Variables	Red River Delta	North Midland & Mountain areas	North Central & Coastal area	Central Highlands	South East	Mekong River Delta
Panel A: Model 1—IMR (%)						
DOC	−0.24	−0.32	−1.00	−0.61	−0.20	−0.27
NUR	−0.05	−0.07	−0.21	−0.12	−0.04	−0.08
MID	−0.73	−0.90	−1.50	−1.17	−0.48	−0.64
PHA	−1.05	−1.40	−4.10	−1.98	−1.77	−0.98
Panel B: Model 2—U5MR (%)						
DOC	−0.37	−0.51	−1.54	−0.96	−0.29	−0.42
NUR	−0.07	−0.11	−0.33	−0.19	−0.06	−0.12
MID	−1.13	−1.40	−2.30	−1.84	−0.70	−0.98
PHA	−1.62	−2.20	−6.29	−3.12	−2.56	−1.51
Panel C: Model 3—LE (years)						
DOC	0.07	0.13	0.39	0.23	0.09	0.12
NUR	0.01	0.03	0.08	0.04	0.02	0.03
MID	0.21	0.34	0.56	0.41	0.21	0.28
PHA	0.29	0.51	1.48	0.68	0.73	0.41

**Table 5:** Estimated impacts of increasing one unit of socioeconomic variables on health outcomes in each region

Variable	Red River Delta	North Midland & Mountain areas	North Central & Coastal area	Central Highlands	South East	Mekong River Delta
Panel A: Model 1—IMR (%)						
IPC (million)	−0.04	−0.18	−0.17	−0.16	−0.05	−0.03
RPD (person/km^2^)	0.00	−0.08	−0.09	−0.06	0.00	−0.03
IR (%)	0.15	0.10	0.44	0.14	0.08	0.12
PR (%)	0.01	0.01	0.02	0.01	0.02	0.02
Panel B: Model 2—U5MR (%)						
IPC (million)	−0.07	−0.31	−0.29	−0.27	−0.07	−0.05
RPD (person/km^2^)	−0.01	−0.12	−0.14	−0.09	0.00	−0.05
IR (%)	0.23	0.16	0.67	0.21	0.12	0.18
PR (%)	0.02	0.02	0.02	0.02	0.03	0.03
Panel C: Model 3—LE (years)						
IPC (million)	0.01	0.07	0.07	0.06	0.02	0.02
RPD (person/km^2^)	0.00	0.03	0.04	0.02	0.00	0.01
IR (%)	−0.04	−0.04	−0.17	−0.05	−0.04	−0.05
PR (%)	0.00	−0.01	−0.01	−0.01	−0.01	−0.01

Note: “” not statistically significant at the 5% level

Four issues are evident in the findings. First, the Wald test of all three models rejects the null hypothesis that all coefficients are equal to zero with *p* value less than 0.05. In other words, our explanatory variables can explain the dependent variables of health outcomes to a significant extent. However, these models explain only around 52, 53, and 64% of variation in IMR, U5DR, and LE, respectively. These results reflect the fact that our models focus on examining only a limited number of inputs of the wider health system, which also comprise other important components such as finance, physical infrastructure, and consumables [41].

Second, life expectancy and mortality rates are statistically associated with density of all four examined categories of workforce (*p* < 0.05). An increase in the number of each of four categories of health workers is likely to have a positive impact on life expectancy in all regions. In our estimation, while holding other variables constant, having one doctor more per 10 000 people on average will add up to 4.12 months to life expectancy. Moreover, Table 4 presents the calculation of the different impact of the density of each category of health workers on health outcomes in each economic region. For instance, the impact of increasing the number of doctors in the Central Highlands has a larger effect on IMR and U5MR than on the Red River Delta and the Southeast regions. There is a big elasticity between the regions, i.e., economically marginal impact of number of doctors on mortality rate and life expectancy is largest in the North Central and Coastal and smallest in the Red River regions. The same trends are in relation to numbers of nurses, midwives, and pharmacists.

Third, although densities of doctors, nurses, midwives, and pharmacists are all negatively related to mortality rates (*p* < 0.05), densities of midwives and pharmacists have stronger impacts on these rates than those of doctors and nurses. It is estimated that, while holding other variables fixed, on average if there are 10 doctors more and 10 nurses more per 10 000 population, the IMR decreases, respectively, by 4.4% (i.e., 44 deaths less per 1 million live births) and 1% (10 deaths less per 1 million live births). Meanwhile, this effect is much bigger for midwives and pharmacists, at 9% (90 deaths per 1 million live births) and 19% (190 deaths per 1 million live births), respectively.^2^ The densities of doctors, nurses, midwives, and pharmacists have the largest effects on IMR and U5MR in North Central and Coastal and the smallest in Southeast regions (see Table 4).

Fourth, income per capita and population density affects positively the life expectancy (*p* < 0.01) and negatively the child and infant mortality rates (*p* < 0.05). Adult illiteracy rate is also associated positively with life expectancy and negatively with child and infant mortality rates (*p* < 0.01).

## Discussion

Since 1990, Vietnam has undergone a variety of health sector reforms. Key reforms included recognition and legalization of the private health care, introduction of the user charges and health insurance, and liberalization of the pharmaceutical market, all leading to the state health budget becoming no longer the only source of finance of Vietnamese health system. These reforms have affected governance and regulation of health workers. And as a result, the government has reduced its subsidy for health workers’ education and it is no longer compulsory for medical graduates to be assigned by the Ministry of Health to their working place and positions. Health workers are now free to choose their preferred working place in the job’s market. This policy has a two-sided effect on the distribution of health workers. On the one hand, this encourages the performance of the health workers to respond to the demand of health markets. On the other hand, the policy leads to imbalanced distribution of health workers across the regions. Although the government has created non-financial and financial incentives to recruit, deploy, and retain health workers in disadvantaged and remote areas, this issue remains a major challenge to address in Vietnam.

During the last few decades, there has been an increasing interest in exploring relations between availability of human resources for health and health outcomes [1]. Given that population’s health outcomes are a product of complex and interdependent interventions, disentangling and weighting this relation with regression models can be informative and useful for policy reforms, to better plan and manage human resources for improving health outcomes.

Our empirical analysis shows a positive impact of the number of health workers on increases in life expectancy and decreases in infant and under-five mortality rates. This finding confirms the importance of availability of health workforce on improving health outcomes in ensuring the achievement of objectives of national health systems reported elsewhere [39, 42, 43]. Moreover, the different impacts of density of each category of health workers on health outcomes in each economic region (Table 4) can inform decisions on where the priorities of investment into human resources should be placed upon to have optimal effect for the whole country.

We found that density of pharmacists is most strongly and statistically linked to life expectancy and mortality rates. Vietnam is currently lacking pharmacy personnel at all levels, and the distribution of their limited numbers is imbalanced between the different regions. Furthermore, the pharmaceutical industry attracts pharmacists better than public hospitals, health centers, and institutions [44]. As a result, the shortage of pharmacists in health facilities is more severe. More importantly, the pharmacy profession is still not appropriately recognized in Vietnam [43, 45]. Yet pharmacists play important roles as counselors and health providers, often by supplying quality information to patients alongside dispensing medicines. Therefore, they should be explicitly considered in the Master Plan for Human Resources Development, aiming at better health outcomes. An important caveat, however, is appropriate here that visiting a pharmacy without consulting a doctor is often not a good practice and therefore should not be promoted particularly for patients with complex health problems such as multiple co-morbidities.

The density of midwives is statistically significant to three key health outcomes and has much more impact on mortality rates and life expectancy than densities of nurses and doctors. Midwifery practice plays a crucial role in reducing child deaths and improving maternal health within Vietnam’s health system [15]. At the primary health care level, where doctors are not always the first point of contact, access to skilled midwives can ensure equitable access to maternal health services particularly to vulnerable groups such as ethnic minorities [46]. Also, the stronger impact of the density of midwives and pharmacists on mortality rates is likely due to the fact that in a developing country like Vietnam, access to midwives and pharmacists is both financially and geographically easier than to other health workers such as doctors and nurses.

The adult illiteracy rate is related positively to life expectancy, and negatively to mortality rates. While the poor social and economic conditions of parents link to poor health, the inability to read and write is also a barrier to acquiring sufficient information for potential self-care. Thus, future research which disentangles this relationship can inform effective and targeted interventions.

Our finding of the relationship of income and health outcomes is consistent with a range of other qualitative and quantitative research in different contexts [47–49]. People from higher income regions on average have better health outcomes. This has an implication for Vietnam’s health system which aims to achieve health equity. As the inequality of income still exists between the different regions of the country, this goal is unlikely to be attainable. Therefore, raising the incomes of the habitants living in the disadvantaged regions should help to mitigate health inequality, and ultimately improve population’s health.

While our analysis specifically focused on availability of different categories of workforce, we also recognize the importance of their performance. The numbers of staff can be high, but poorly performing workforce may not add sufficient gains to life expectancy and reduction to mortality rates. Conversely, even if staff numbers are smaller, improvements in their performance (for example, through improving supportive supervision and performance appraisal and introducing incentives such as performance-based payments) may help achieve substantial improvements in health outcomes. Therefore, any policy interventions related to human resources for health should recognize both the importance of staff availability and their quality or performance.

Finally, although health workforce numbers and their performance are clearly important, the general development, such as the earlier-discussed income and education levels, is likely to also have positive implications on achievement of improved health outcomes, perhaps even more significant than availability of individual components of the health systems [50, 51]. Therefore, effective interventions for improving health outcomes need to focus on more integrated development, building on the well-recognized link between health and development, rather than focus on health care sector in relative isolation.

### Study limitations

In our models, we assumed that all doctors have the same qualification and competence levels. However, qualifications and competences of doctors can be different across and within the different economic regions, which can ultimately determine their performance. Although exploring performance of health workers was outside the scope of our study, this can be a potential question for further research in Vietnam. Our model examined only four main categories of health workforce. Meanwhile, other types of health workers such as dentists, technicians, and managers are also likely to contribute to achievement of health outcomes. Our decision to focus on these four categories was driven by our experience, which shows that these four generic categories can be found in most health systems, thus contributing to the relevance of our results to different contexts. We did not account for the difference of urban and rural distribution of health workforces whose density is likely higher at urban areas and is influenced by political and economic factors. Again, this can be a potential question for future studies.

## Conclusions

This study statistically examined the relationship between availability of health workers and health outcomes in Vietnam. Our results suggest that availability of four main categories of health workers can contribute to achieving better health outcomes and ultimately expending the life expectancy of populations, underlining the importance of investing in health workforce in strengthening national health systems. Therefore, increasing investment into more equitable distribution of human resources for health, with focus on four main categories of workforce (doctors, nurses, midwives, and pharmacists) represents an important strategy for improving health outcomes, while we also recommend that future interventions will need to consider an integrated development approach, building on the link between health and development.

## Additional file

Additional file 1: Supplementary material presented in Appendices 1-5. (DOC 349 kb)

## Abbreviations

DOC: Doctor density
IMR: Infant mortality rate
IPC: Income per capita
IR: Adult illiteracy rate
LE: Life expectancy at birth
MID: Midwife density
NUR: Nurse density
PCA: Principal component analysis
PHA: Pharmacist density
PR: Poverty rate
RPD: Regional population density
U5MR: Under-five mortality rate

## Footnotes

1 The government decree number 92/2006/NĐ-CP on the establishment, approval, and management of the master plan on socioeconomic development dated 7 November 2006 divided Vietnam into six economic regions which are special zones of national economy and in which particular types of commerce and manufacturing take place based on geographical boundaries.

2 To calculate the average effect, we averaged all estimated coefficients from Table 4 over six regions, and converted to the corresponding measurement units.
