# Supplementary material for: Contribution of health workforce to health outcomes: empirical evidence from Vietnam
Source: Hum Resour Health. 2016 Nov 16;14:68. doi: 10.1186/s12960-016-0165-0 (PMC5112617; doi:10.1186/s12960-016-0165-0)
Supplement: Supplementary file 1 — Supplementary material presented in Appendices 1-5. (DOC 349 kb) [file 12960_2016_165_MOESM1_ESM.doc]

# APPENDICES

Appendix 1: Multicolinearity test

The variance inflation factors (VIF) is used as an indicator of correlation among explanatory variables (EVs).

### Table A1: Results of correlations of explanatory variables

| Explanatory variables | VIF |
| --- | --- |
| PR | 33.91 |
| POP | 20.95 |
| IPC | 19.10 |
| MID | 9.32 |
| DOC | 8.35 |
| IR | 7.27 |
| NUR | 5.58 |
| PHAR | 3.99 |

As recommended by Rogerson, P. A. (2001), the value of VIF more than 5 is considered as sign of serious collinearity.

Appendix 2: The principal component analysis (PCA)

## Step 1: Standardize variables

As each variable has its own unit of measurements (number, percentage) yet our goal is to weigh the relative importance of each variable on health outcomes, we need to standardize those variables to make them compatible for that purpose. All variables are standardized accordingly to the following formula


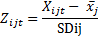


Where

X*_ijt_* = Data for variable *_j_* in region it time *_t_*


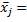
 Mean for variable *_j_* in region *_i_* over time

SD*_ij_*= Standard deviation for variable *_j_* in region *_i_*

## Step 2: Implement PCA on standardized explanatory variables

Then we perform the Principal Component Analysis on the observed data matrix of EVs to determine the number of principal components by computing their eigenvalues and by looking at a Scree Plot for standardized variables. Those eigenvalues and their proportional and cumulative contribution to the variation of data are presented in Table A2, and the Scree Plot in Figure A1.

### Table A2: Results from PCA of explanatory variables

| Component | Eigenvalue | Proportion | Cumulative |
| --- | --- | --- | --- |
| 1 | 5.58 | 0.78 | 0.78 |
| 2 | 0.70 | 0.10 | 0.88 |
| 3 | 0.36 | 0.05 | 0.93 |
| 4 | 0.20 | 0.03 | 0.96 |
| 5 | 0.10 | 0.01 | 0.97 |
| 6 | 0.08 | 0.01 | 0.98 |
| 7 | 0.08 | 0.01 | 0.99 |
| 8 | 0.05 | 0.01 | 1.00 |

### Figure A1: Scree plot of eigenvalues

As Table A2 shows, the proportion of variation explained by each eigenvalue is given in the third column. The cumulative column shows 88% of the variation of EVs explained by the first two components. According to Cattell‘s (1966), we retain the 2 first components (PC1, PC2) which have the biggest eigenvalues and explain most of variation in EVs.

Corresponding to the eigenvalues are their eigenvectors as shown in Table A3

### Table A3: The scores of the first two principal components

| EVs (standardized) | Eigenvector correspond to  eigenvalue of Component 1 | Eigenvector correspond to  eigenvalue of Component 2 |
| --- | --- | --- |
| DOC | 0.33 | 0.01 |
| NUR | 0.38 | -0.12 |
| MID | 0.38 | 0.18 |
| PHAR | 0.24 | 0.88 |
| IPC | 0.39 | -0.13 |
| POP | 0.38 | -0.19 |
| PR | -0.34 | 0.37 |
| IR | -0.38 | 0.02 |

Thus, the scores of the first two principal components are constructed as the following formula

PC*i* = e*_i_* * Z*_ijt_*

where

e*_i_* is the corresponding eigenvectors

Z*_ijt_* is the standardized variable *_j_* in region *_i_* time *_t_*

PC*_i_* is the principal component *_i_*

More particularly, the principal components PC*_1_* and PC*_2_* are

PC*_1_*=0.33*DOC+0.38*NUR+0.38*MID+0.24*PHA+0.39*IPC+0.38*RPD-0.34*PR-0.38*IR

PC*_2_*=0.01*DOC-0.12*NUR+0.18*MID+0.88*PHA-0.14*IPC+0.14*RPD+0.37*PR+0.02*IR

## Step 3: Use an appropriate Principle Component Regression (PCR) which accounts for both cross-section dependence and serial correlations

We now can use PC*_1_*, PC*_2_* in our PCR regression as predictors for health outcomes as below

Y_it_ = *a*_0_* Time + *a*_1_*PC1 + *a*_2_*PC2 +c*_0_* + e*_it_*

where

*a*_0_*, a*_1_*, a*_2_ are the coefficients of the principal components

e_it_ are the regression errors.

The value of estimated coefficients is reported at table A4.

### Table A4: Result of the estimated coefficients of PCR

|  | Model 1: IMR | Model 2: U5M | Model 3: LE |
| --- | --- | --- | --- |
| *a*_0_ | -0.45 | 0.45 | 0.50 |
| *a*_1_ | -0.36 | 0.37 | 0.38 |
| *a*_2_ | 2 | 2 | -2 |

## Step 4: Recover the interested coefficients of our main models

Finally, the coefficients
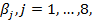
 of our models are recovered from the estimates *a*_0_*, a*_1_*, a*_2_ of the above PCR as described in the following process.

Let *W_k_* (*k*=1,2) is data matrix of (PC_1_, PC_2_), and *V_k_* is a matrix of their corresponding eigenvector (e_1_, e_2_). Let
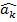
= (
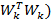
-1
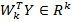
 denote the vector of estimated coefficients obtained by PCR of health outcomes. Then the estimates of
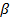
, i, e,
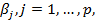
 in our model by using PCR based on the first two principal components PC_1_, PC_2_ is given by:
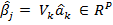
 (*p*=8: the number of coefficients need estimating). We then recover the standard errors of
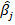
 accordingly.

Appendix 3 :Table A5: Tests for Cross-Section Dependence and Serially Correlated Errors in PCR

| Explanatory variables | Model 1:IMR | Model 2:U5MR | Model 3:LE |
| --- | --- | --- | --- |
| PC1 | -0.42  (0.10) | -0.43  (0.10) | 0.45  (0.10) |
| PC2 | -0.37  (0.16) | -0.37  (0.16) | 0.40  (0.15) |
| Time | 1.97  (0.49) | 1.97  (0.48) | -1.80  (0.47) |
| Constant | -1.23  (0.34) | -1.23  (0.34) | 1.12  (0.39) |
| N | 48 | 48 | 48 |
| R^2^ overall | 0.34 | 0.34 | 0.413 |
| F | 6.68 | 6.80 | 1.540 |
| Pro>χ^2^ | 0.00 | 0.00 | 0.00 |
| Free’s test | 0.66 | 0.47 | 0.19 |
| Wooldridge test | 196.52 | 9.41 | 9.35 |

Note: The number in parenthesis is the standard error

The Frees (1995, 2004) test for cross-sectional dependence is a post estimation test of the above panel regression with fixed effect. The test is aimed at checking if the errors are correlated across regions. This cross-sectional dependence can lead to bias estimation of the models. The null hypothesis H_0_: there is no correlation of errors across regions. And the critical values for Free’s Q distribution at 1%, 5% and 10% significance level are 0.66, 0.43, and 0.32 correspondingly. For all three models, the Free’s test does reject the H_0_ at 5% significant level, i.e, the test implies that cross-sectional dependence is available within our panel of 6 regions.

The Wooldridge test is aimed to check if the error terms of time periods are correlated. The null hypothesis is that errors in one time period are not correlated with the errors ensuing time period (first-order correlation). For all three models, the Wooldridge tests reject the null hypothesis. In other words, there is evidence that the errors are serially correlated of order 1.

Appendix 4

### Table A6: Estimated impacts of increasing 1 health worker per 10,000 population on health outcomes in each region

| **Variables** | Red  River  Delta | North  Midland&  Mountain areas | North  Central &  Coastal area | Central Highland | South East | Mekong River Delta |
| --- | --- | --- | --- | --- | --- | --- |
|  |  |  | |  |  |
|  |  |  |  |  |  |  |
| **Panel A: Model 1 - IMR (%)** | | |  |  |  |  |
| DOC | -0.24 | -0.32 | -1.00 | -0.61 | -0.20 | -0.27 |
| NUR | -0.05 | -0.07 | -0.21 | -0.12 | -0.04 | -0.08 |
| MID | -0.73 | -0.90 | -1.50 | -1.17 | -0.48 | -0.64 |
| PHA | -1.05 | -1.40 | -4.10 | -1.98 | -1.77 | -0.98 |
|  |  |  |  |  |  |  |
| **Panel B: Model 2 - U5MR (%)** | | |  |  |  |  |
| DOC | -0.37 | -0.51 | -1.54 | -0.96 | -0.29 | -0.42 |
| NUR | -0.07 | -0.11 | -0.33 | -0.19 | -0.06 | -0.12 |
| MID | -1.13 | -1.40 | -2.30 | -1.84 | -0.70 | -0.98 |
| PHA | -1.62 | -2.20 | -6.29 | -3.12 | -2.56 | -1.51 |
|  |  |  |  |  |  |  |
| **Panel C: Model 3 - LE (years)** | | |  |  |  |  |
| DOC | 0.07 | 0.13 | 0.39 | 0.23 | 0.09 | 0.12 |
| NUR | 0.01 | 0.03 | 0.08 | 0.04 | 0.02 | 0.03 |
| MID | 0.21 | 0.34 | 0.56 | 0.41 | 0.21 | 0.28 |
| PHA | 0.29 | 0.51 | 1.48 | 0.68 | 0.73 | 0.41 |

Appendix 5

### Table A7: Estimated impacts of increasing 1 unit of socio-economic variables on health outcomes in each region

| Variable | Red River Delta | North Midland  &  Mountain areas | North Central &  Coastal area | Central Highland | South East | Mekong River Delta |
| --- | --- | --- | --- | --- | --- | --- |
|  |  |  |  | |  |  |
|  |  |  |  |  |  |  |
| **Panel A: Model 1 - IMR (%)** | |  |  |  |  |  |
| IPC (Million) | -0.04 | -0.18 | -0.17 | -0.16 | -0.05 | -0.03 |
| RPD (person/km2) | 0.00 | -0.08 | -0.09 | -0.06 | 0.00 | -0.03 |
| IR (%) | 0.15 | 0.10 | 0.44 | 0.14 | 0.08 | 0.12 |
| PR (%) ⱡ | 0.01 | 0.01 | 0.02 | 0.01 | 0.02 | 0.02 |
|  |  |  |  |  |  |  |
| **Panel B: Model 2 - U5MR (%)** | | |  |  |  |  |
| IPC (Million) | -0.07 | -0.31 | -0.29 | -0.27 | -0.07 | -0.05 |
| RPD (person/km2) | -0.01 | -0.12 | -0.14 | -0.09 | 0.00 | -0.05 |
| IR (%) | 0.23 | 0.16 | 0.67 | 0.21 | 0.12 | 0.18 |
| PR (%) ⱡ | 0.02 | 0.02 | 0.02 | 0.02 | 0.03 | 0.03 |
|  |  |  |  |  |  |  |
| **Panel C: Model 3 - LE (years)** | | |  |  |  |  |
| IPC (Million) | 0.01 | 0.07 | 0.07 | 0.06 | 0.02 | 0.02 |
| RPD (person/km2) | 0.00 | 0.03 | 0.04 | 0.02 | 0.00 | 0.01 |
| IR (%) | -0.04 | -0.04 | -0.17 | -0.05 | -0.04 | -0.05 |
| PR (%) ⱡ | 0.00 | -0.01 | -0.01 | -0.01 | -0.01 | -0.01 |

Note: " ⱡ " not statistically significant at 5% level
